# Integrated Timing of Stroking, Breathing, and Kicking in Front-Crawl Swimming: A Novel Stroke-by-Stroke Approach Using Wearable Inertial Sensors

**DOI:** 10.3390/s22041419

**Published:** 2022-02-12

**Authors:** Silvia Fantozzi, Vittorio Coloretti, Maria Francesca Piacentini, Claudio Quagliarotti, Sandro Bartolomei, Giorgio Gatta, Matteo Cortesi

**Affiliations:** 1 Department of Electrical, Electronic, and Information Engineering “Guglielmo Marconi”, University of Bologna, Viale Risorgimento 2, 40136 Bologna, Italy; vittorio.coloretti@studio.unibo.it; 2 Health Sciences and Technologies-Interdepartmental Centre for Industrial Research, University of Bologna, Viale Risorgimento 2, 40136 Bologna, Italy; 3 Department of Movement, Human and Health Sciences, University of Rome Foro Italico, 00135 Rome, Italy; mariafrancesca.piacentini@uniroma4.it (M.F.P.); c.quagliarotti@studenti.uniroma4.it (C.Q.); 4 Department of Biomedical and Neuromotor Sciences, University of Bologna, 40126 Bologna, Italy; sandro.bartolomei@unibo.it; 5 Department for Life Quality Studies, University of Bologna, 40126 Bologna, Italy; giorgio.gatta@unibo.it

**Keywords:** kinematics, wearable device, inertial measurement unit, gyroscope, validation, performance analysis, training monitoring

## Abstract

Quantitative evaluation of synergic action among the different body segments is fundamental to swimming performance. The aim of the present study was to develop an easy-to-use tool for stroke-by-stroke evaluation of a swimmer’s integrated timing of stroking, kicking, and breathing. Twelve swimmers were evaluated during one trial of 100 m front-crawl swimming at self-selected speed. Five three-axial inertial sensors were mounted on the head, wrists, and ankles. Algorithms for the wrist entry into the water, the lower limb beat during the downward action, and the exit/entry of the face from/into the water were developed. Temporal events identified by video-based technique, using one sagittal moving camera, were assumed as the gold standard. The performance was evaluated in terms of the root-mean-square error, 90th percentile of absolute error, coefficient of variation, Bland–Altman plots, and correlation analysis. Results of all temporal events showed high agreement with the gold standard, confirmed by a root-mean-square error of less than 0.05 s for absolute temporal parameters and less than 0.7% for the percentages of the stroke cycle duration, and with correlation coefficients higher than 0.856. The protocol proposed was not only accurate and reliable, but also user-friendly and as unobtrusive as possible for the swimmer, allowing a stroke-by-stroke analysis during the training session.

## 1. Introduction

Swimming performance is strictly related to the synergic action among the different body segments. The analysis of a singular body segment does not completely characterize the actions, and the movement of the limbs cannot be considered independently [1,2]. Depending on swimming speed and athletic skill, swimmers change their coordination between limbs and breathing action [3]. Furthermore, the variability characterization of the synergic action is a way to understand the performance profile [4]. Skilled swimmers seem able to maintain a stable pattern of the stroke parameters to standardize their motor pattern and postpone a technique degradation, and conversely in less experienced swimmers [5].

Quantitative evaluation of synergic action and variability is fundamental to provide reliable information to coaches and athletes. Since 1970, video cameras have been exploited for swimmer kinematics analysis from different points of view [6,7,8,9]: timing and distance evaluation, velocity of center of mass, 2D/3D coordinates of anatomical landmarks, and 2D/3D joint angular kinematics. However, the use of multicamera systems requires long processing times; therefore, data are not immediately available. Moreover, the expensive and cumbersome camera setup with a restricted acquisition volume made this technology not well-suited for real-time training context. Since 2000, thanks to the development of wearable inertial sensors, these limits were overcome and ecological evaluation was made available by recording stroke-by-stroke data over a long period [10].

Considering front-crawl, the main contribution for force exertion in the water is attributed to the upper limbs [11,12]. The most commonly estimated quantities for the upper limbs using inertial measurement units (IMUs) were stroke count and stroke rate, achieved by locating the sensors on the wrist or/and back and applying a processing algorithm on raw data, with an accuracy between 65% and 99% [13,14,15]. The temporal segmentation of the different phases (entry and catch, pull, push, recovery) within the stroke was achieved: (i) using specific features directly on the raw data of the wrist sensors [16]; (ii) from three sensors positioned on both forearms and lower back [17]; or (iii) using the arm kinematic chain for estimating the joint angular kinematics from the orientation of five sensors on the trunk, arms, and forearms [18,19]. Despite its key role within the stroke, the entry event is the most critical instant to be detected by IMU, probably due to high variation related to the swimmer specialization [20,21]. From temporal-phase parameters, together with position and velocity of body segments, previous studies investigated start/end propulsion of one arm with respect to the other (coordination index) [17], the interlimb coordination (continuous relative phase) [22], and synchronization [23]. The adaptive role of movement and coordination variability were assessed by using these motor control indexes, highlighting how behavior functionally responds to environmental and task constraints [4,24].

The action of the lower limbs is essential not only for trunk balance and buoyancy [11], but also influences the arms’ propulsive action by modifying the hand trajectory and by increasing stroke length [25]. Regarding flutter kicking, the main estimated parameters using IMU were kick count and kick rate, showing reliable results (standard error of the estimate: 5.9 ± 0.5%) [26] and allowing an evaluation of the fatigue effect on this parameter [27]. In both articles, specific features drawn directly from the raw data of the ankle sensor were exploited [26]. However, the synergic action of the lower limbs with respect to the upper limbs or its variability were not investigated.

The breathing frequency or laterality was investigated as an independent variable to estimate the effect on stroke parameters [28]. However, no specific analysis regarding breathing technique was performed using IMUs. The sensor location on the head had several advantages, as it did not affect drag, and measured overall body motion. For this reason, this location was used to estimate the stroke parameters or intracyclic velocity of the swimmer [29,30]. Nevertheless, Shell et al. found the accuracy of stroke parameter estimation was not sufficient enough for the training monitoring tool [29].

As previously highlighted, the accuracy of temporal parameters for upper and lower limb actions were investigated independently. To give a complete characterization of the swimmer’s action and to understand the coordination between the movements of the head and the upper and lower limbs, the combination of these features should be taken into account. Nevertheless, a combined timing analysis of stroking, kicking, and breathing actions has not been evaluated. Furthermore, this type of analysis must be performed in ecological conditions, and the protocol should be friendly and plain enough to be used by coaches during training sessions. For this reason, the aim of the present study was to develop and validate an easy-to-use tool for stroke-by-stroke evaluation of a swimmer’s integrated timing of stroking, kicking, and breathing. The validation of temporal parameter estimation for the upper limbs, the lower limbs, and the breathing action were performed singularly. We hypothesized that IMU technologies would allow the estimation of swimmer timings regarding stroking, kicking, and breathing with sufficient accuracy for the purpose.

## 2. Materials and Methods

### 2.1. Protocol: Participants, Trials and Instrumentation

Twelve male swimmers (age: 19.1 ± 2.3 years; mass: 76.7 ± 3.7 kg; height: 179.0 ± 5.2 cm; level: 686 ± 82.6 FINA points long course, Tier 3 [31]) were evaluated during front-crawl swimming at self-selected speed in a 25 m swimming pool. For each participant, one trial of 100 m was acquired.

**Figure 1:**
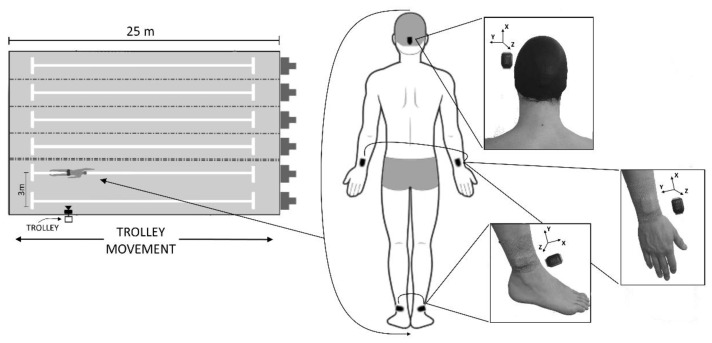
Single sagittal camera on trolley followed the athlete at similar velocity during 100 m front-crawl swimming (**left**). Positioning of wearable inertial sensors on head, wrist, and ankle (**right**). Alignment of axes (*X*, *Y*, and *Z*) of reference system are shown for each position.

Five triaxial IMUs (Cometa, Milano, Italy) equipped with an accelerometer (sensitivity: 1563 mV/g; full scale: ±16 g) and gyroscope (sensitivity: 1.3 mV/g; full scale: ±2000°/s) were calibrated at the beginning of each acquisition session. Successively, IMUs were attached to the head (in the occipital zone between the supreme and superior nuchal lines), forearms (about two centimeters above the styloid), and shanks (about two centimeters above lateral malleolus). Then, 3D acceleration and 3D angular velocity were acquired from each sensor with a sampling frequency of 285 Hz. Biadhesive tape (0.05 m × 25 m Eurocel, SICAD S.p.A. ITALIA, Varese, Italy) was used for all IMUs. In addition, for the head, the sensor was inserted between two swim caps, and for the limbs, a coband (BSN Medical Co-Plus^®^ Lf 0.075 m × 4.5 m, BSN medical GmbH, Hamburg, Germany) was used to firmly fix the sensors. The IMU sensor for the head had the *X*-axis aligned with the longitudinal axis of the skull pointing upward, the *Y*-axis with the transverse axis pointing to the left, and the *Z*-axis consequently aligned. The IMU sensor for the wrist had the *X*-axis aligned with the longitudinal axis of the forearm pointing proximally, the *Y*-axis with the radioulnar axis, and the *Z*-axis consequently aligned. The IMU sensor for the ankle was aligned with the *X*-axis aligned to the sagittal axis of the tibia pointing forward, the *Y*-axis to the longitudinal axis pointing proximally, and the *Z*-axis consequently aligned. Details of the IMU attachment and axes alignment are reported on the right in Figure 1.

The swimming trials were also filmed using a single moving sagittal video camera (Hero7, GoPro, San Mateo, CA, USA, sf = 240 Hz, 1920 × 1080 pixel resolution) for the event detection gold standard (TLC). The camera was held on a sagittal plane by an operator following the swimmer at similar velocity using a trolley (Figure 1 on the left). Stroking, breathing, and kicking temporal events were clearly visible on the images of the video for all the strokes within the lap. The same expert operator identified all the events in the video. The sensors’ flashing LEDs were video-recorded to synchronize data acquired using IMUs and video recording.

### 2.2. Data Analysis

#### 2.2.1. Stroking

**Figure 2:**
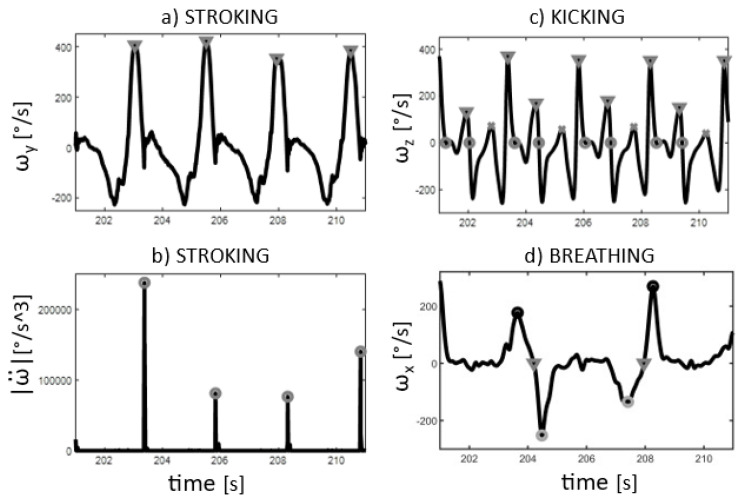
A typical pattern of four strokes for one swimmer. Stroking events using the wrist IMU: (**a**) angular velocity of Y axis with maximum for recovery phase identification (grey triangle); (**b**) modulus of the angular jerk with maxima for WRIST_ENTRY_ (grey circles). Kicking events using the ankle IMU: (**c**) angular velocity of *Z*-axis with propulsive (grey triangle) and buoyancy (grey cross) kicking, and zero-crossing for LEG_DOWNBEAT_ (grey circles). Breathing events using the head IMU: (**d**) angular velocity of *X*-axis with zero-crossing (grey triangle) between maximum/minimum (black/grey circle) for HEAD_EXIT_ and HEAD_ENTRY_ (before and after zero-crossing maximum/minimum events, respectively).

In the present analysis, the entry of the wrist into the water (WRIST_ENTRY_) was used for the identification of the stroke cycles. The WRIST_ENTRY_ was estimated from the modulus of the angular jerk measured by the wrist sensor. The modulus was preferred with respect to a specific axis in order to be as independent as possible from the specific swimmer’s technique. First, a clearly identifiable instant of the recovery phase was detected by calculating the maximum value of the angular velocity about the radioulnar direction (*Y*-axis). Then, the modulus of the angular jerk was calculated by performing the double differentiation of the wrist angular velocity. Finally, the WRIST_ENTRY_ event was determined as the maximum of the jerk modulus between two previously identified successive recovery phases. An example pattern of the quantities used for the algorithm and the estimated WRIST_ENTRY_ event are given in Figure 2a,b, respectively.

#### 2.2.2. Kicking

For the time characterization of the leg action, the event corresponding to the beat of the lower limb during the downward action (LEG_DOWNBEAT_) was considered. The mediolateral angular velocity of the IMUs on the ankle was used (*Z*-axis). To estimate the beat, the zero-crossing of the angular velocity was employed, and to distinguish the down- from the up-beat, the orientation of the axis and the sign before and after the zero-crossing were taken into account. An example pattern of the quantities used for the algorithm and the estimated LEG_DOWNBEAT_ event is given in Figure 2c.

In the analysis, two types of kicking actions were observed: propulsive and buoyancy kicks. They were first visually inspected considering the regularity of the leg movement: in some swimmers, the pattern of the angular velocity had similar maximum values throughout the trials, while others showed two/three maximum comparable values followed by one at a clearly distinguished lower maximum value. This last beat was interpreted as a beat performed for buoyancy purposes and not for propulsion. For this reason, this maximum was not included in the analysis: an adaptable threshold was calculated from the mean plus two standard deviations of all maxima below 100 deg/s of each athlete. The starting value of 100 deg/s and the results of the exclusion were established from the video analysis, in which the difference between propulsive and buoyancy kicking was clearly distinguished.

#### 2.2.3. Breathing

Regarding the breathing analysis, the exit and the entry of the face from/into the water were considered (HEAD_EXIT_ and HEAD_ENTRY_, respectively). The longitudinal angular velocity of the IMUs on the head was used (*X*-axis). From the zero-crossing of this variable, the start of the face rotation out of the water was first identified. Successively, the maximum/minimum values before and after the zero-crossing corresponded to the exit/entry of the head depending on the breathing side: the event before the zero-crossing was the exit, the event after was the entry. An example pattern of the quantities used for the algorithm and of the estimated HEAD_EXIT_ and HEAD_ENTRY_ events is given in Figure 2d.

#### 2.2.4. Timing

From the left WRIST_ENTRY_, the 0% and the 100% of the stroke cycle were determined. WRIST_ENTRY_ contralateral arm, LEG_DOWNBEAT_, HEAD_EXIT_, and HEAD_ENTRY_ events were expressed in percentage of the stroke cycle.

Data processing of the three above algorithms and timing analysis were performed in MATLAB (MathWorks, Natick, MA, USA, 2019).

#### 2.2.5. Statistical Analysis

All data were expressed as means (±SD). The Shapiro–Wilk test was used to confirm the normality of distribution. Regarding the WRIST_ENTRY_, HEAD_EXIT_, HEAD_ENTRY_, and LEG_DOWNBEAT_ absolute temporal values and percentages of the stroke cycle, the accuracy of the IMU algorithms in comparison to TLC were determined by the root-mean-square error (RMSE [32]) and 90th percentile of absolute error, and the reliability was computed using the coefficient of variation (CV) and typical error of measurement (TEM). Considering the sampling frequency of the video acquisition, a minimum detectable difference threshold of 0.005 s was considered. To complement the agreement analyses between the two techniques, Bland–Altman plots and a correlation analysis were created. To calculate the concurrent validity, the bivariate Pearson product moment correlation coefficient was used. The correlation magnitude was interpreted as 0.1 (low), 0.3 (moderate), 0.5 (large), 0.7 (very high), and 0.9 (nearly perfect) as proposed by Hopkins et al. [33]. All statistical tests were performed using the software SPSS version 20.0 (SPSS, Chicago, IL, USA) and Microsoft Excel 2010. The level of statistical significance was set at *p* < 0.05.

## 3. Results

Regarding absolute temporal variables, the average RMSEs and 90th percentile of absolute errors for IMU vs. TLC were <0.005 s and 0.041 s for WRIST_ENTRY_, 0.005 s and 0.197 s for HEAD_EXIT_, <0.005 s and 0.060 s for HEAD_ENTRY_, and <0.005 s and 0.040 s for LEG_DOWNBEAT_. The CVs for IMU and TLC were 0.068 and 0.069 for WRIST_ENTRY_, 0.440 and 0.200 for HEAD_EXIT_, 0.070 and 0.062 for HEAD_ENTRY_, and 0.209 and 0.204 for LEG_DOWNBEAT_. The respective TEMs for IMU and TLC were 0.083 and 0.083 s for WRIST_ENTRY_, 0.147 and 0.085 s for HEAD_EXIT_, 0.081 and 0.073 s for HEAD_ENTRY_, and 0.240 and 0.232 s for LEG_DOWNBEAT_.

**Figure 3:**
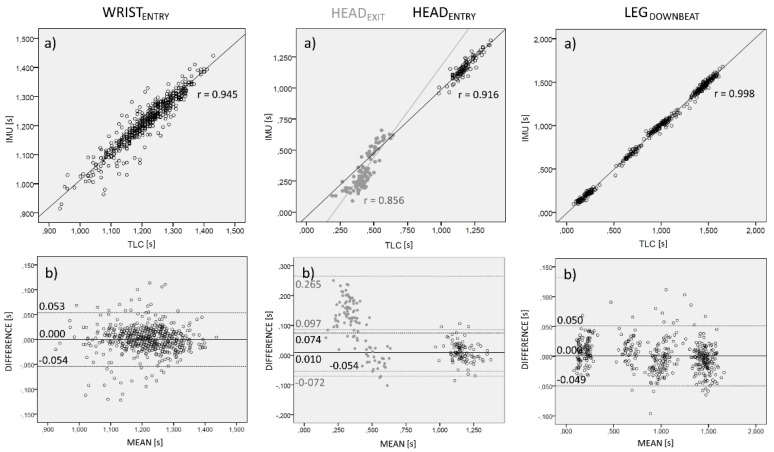
Linear regression analysis (**a**) and Bland–Altman plots (**b**) for WRIST_ENTRY_ (open circles), HEAD_EXIT_ (grey filled circle), HEAD_ENTRY_ (open circle), and LEG_DOWNBEAT_ (open circle) when comparing measurements with IMUs and video-based technique (TLC). In (**b**), central lines represent the intermethod differences (biases). Upper and lower dotted lines represent the 95% limits of agreement (bias ± 1.96 SD of the differences).

Bland–Altman and correlation procedures were employed to determine measurement bias (Figure 3). The limits of agreement (LoA) between the IMU and TLC had a range of −0.054 to 0.053 s for WRIST_ENTRY_, −0.072 to 0.265 s for HEAD_EXIT_, −0.054 to 0.074 s to for HEAD_ENTRY_, and −0.049 s to 0.050 s for LEG_DOWNBEAT_ with nonsignificant biases of <0.005 s, 0.097 s, 0.010 s and <0.005 s, respectively. In addition, the Pearson product moment correlation coefficient showed nearly perfect correlation between the IMU and TLC measurements for WRIST_ENTRY_ (r = 0.945, *p* < 0.001), HEAD_EXIT_ (r = 0.856, *p* < 0.001), HEAD_ENTRY_ (r = 0.916, *p* < 0.001), and LEG_DOWNBEAT_ (r = 0.998, *p* < 0.001). In general, IMU algorithms showed consistent results for all analyzed temporal events.

**Figure 4:**
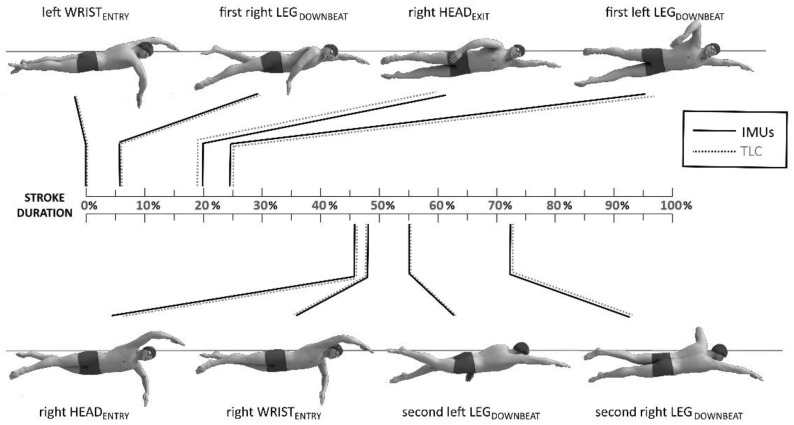
The timing of the analyzed temporal events in percentage of the stroke duration using IMUs (solid black lines) and video-based technique (TLC, dotted grey lines) for a single participant. The stroke duration was normalized to left WRIST_ENTRY_ events. The ticks in the stroke duration axis correspond to 5%.

The average timing of the analyzed temporal events in percentage of the stroke duration are reported in Table 1. Regarding timing, the respective average RMSEs and 90th percentile of absolute errors for IMU vs. TLC were 0.1 and 1.5% for WRIST_ENTRY_, 0.7 and 8.0% for HEAD_EXIT_, 0.1 and 2.2% for HEAD_ENTRY_, and 0.1 and 1.8% for LEG_DOWNBEAT_. The respective CVs for IMU and TLC were 0.0 and 0.0 for WRIST_ENTRY_, 0.4 and 0.1 for HEAD_EXIT_, 0.0 and 0.0 for HEAD_ENTRY_, and 0.1 and 0.1 for LEG_DOWNBEAT_. The respective TEMs for IMU and TLC in percentage of the stroke duration were 2.2 and 2.1 for WRIST_ENTRY_, 4.6 and 3.2 for HEAD_EXIT_, 1.5 and 1.3 HEAD_ENTRY_, and 2.0 and 1.6 for LEG_DOWNBEAT_. The mean biases of timing between measurement techniques were −0.2% for WRIST_ENTRY_ (LoA: −2.3 to 1.9%), 4.1% for HEAD_EXIT_ (LoA: −3.1 to 11.2%), 0.3% for HEAD_ENTRY_ (LoA: −1.9 to 2.5%) and 0.5% for LEG_DOWNBEAT_ (LoA: −1.4 to 2.3%). The timings of the analyzed temporal events in percentage of the stroke duration using IMUs and TLC for a single participant are reported in Figure 4.

## 4. Discussion

A complete characterization of a swimmer’s action requires not only an independent analysis of temporal parameters for the head and upper/lower limb actions, but also an integration of these features. Furthermore, the assessment must be performed for the whole motor task and not only for a few strokes. The present study developed an integrated stroke-by-stroke tool for the assessment of the stroking, kicking, and breathing timing in front-crawl swimming using wearable inertial sensors. To test our hypothesis, data for 12 athletes were collected using IMUs attached to the head, wrists, and ankles, and timing features were compared with video analysis. Results of all temporal events showed high agreement with the gold standard video-based technique, confirmed by an RMSE less than 0.05 s for absolute temporal parameters and less than 0.7% for the percentage of the stroke duration.

The WRIST_ENTRY_ event, detected from the jerk of the angular velocity of the IMU on the wrist, showed high agreement with TLC both in terms of absolute temporal value (bias: <0.005 s, LoA: −0.0054 s to 0.053 s) and in terms of percentage of stroke cycle duration (bias: −0.2%, LoA: −2.3% to 1.9%) with nearly perfect correlation (r = 0.945). The present study’s results were comparable with, and for some parameters, even more accurate than those of previous studies. In more detail, when arm kinematic chain and multiple IMUs were exploited, a bias lower than 1.4%, a 95% LoA lower than 7.7%, an RMSE lower than 3.5%, and an r higher than 0.81 were found for entry, catch, pull, push, and recovery phases [19]. When three IMUs and raw data sensors were used, the 95% LoA for stroke phase durations was always lower than 7.9% of the stroke cycle duration [17]. Furthermore, differently from previous studies, the proposed method did not require any specific body segment alignment calibration or any anthropometric measurement, such as the body segment length, and involved the use of only one sensor per limb. In this context, previous studies highlighted how the hand entry was highly dependent on the swimmer’s technique: palmar–dorsal acceleration can exhibit a high or near-zero value depending on a flatter or sharper pitch angle of the hand entry [16]. For this reason, in the developed algorithm, the modulus of the jerk was used while taking into account all three components of the physical quantity. From the data acquired, every single stroke was automatically detected by the present algorithm without any operator supervision.

The algorithm for the analysis of the kicking timing of the lower limbs exhibited high agreement (bias: <0.005 s, LoA: −0.049 s to 0.050 s; bias: 0.5%, LoA: −1.4 s to 2.3 s) and nearly perfect correlation (r = 0.998) with the video-based technique. The algorithm proposed by Fulton et al. for counting the kicks [26] was slightly modified for the estimation of timing in absolute temporal value and in percentage of the stroke cycle. The modification was necessary, as two types of kicking action were noticed in our study participants. Together with the propulsive kicks, clearly distinguishable buoyancy kicks were observed in the videos. In this latter case, the lower limb of the swimmer exhibited a small beat, mainly due to the rotation of the trunk/pelvis segment and not to a strong action of the leg. From observing the angular velocity of the IMU on the ankle, the buoyancy kicks clearly showed lower peak values, and the modification was developed to exclude them from the analysis. These issues were not reported by Fulton et al., most likely because, differently from the present study, maximal-effort trials were acquired [26,27]. No comparison with existing literature regarding timing features could be performed, as the only previous study that exploited IMUs for kicking action analysis investigated the performance of the algorithms only for counts and rate.

The breathing features investigated showed different levels of accuracy depending on the type of event detected. The HEAD_ENTRY_ showed high agreement with video-based techniques both in terms of absolute temporal quantity (bias: 0.01 s; LoA: −0.054 s to 0.074 s) and in terms of percentage of the stroke duration (bias: 0.5%; LoA: −1.9 s to 2.5 s), with nearly high correlation coefficient (r = 0.916, *p* < 0.001). In addition, the HEAD_EXIT_ event detection exhibited a good degree of agreement. In more detail, the larger bias found (0.097 s; 4.1%) with respect to HEAD_ENTRY_ could be explained by a fixed temporal shift between the feature extracted from the longitudinal angular velocity and the exit of the face from the water identified in the video. Furthermore, the LoAs of HEAD_EXIT_ showed higher values (−0.072 s to 0.027 s; −3.1% to 11.2%) and slightly less reliability for the analysis of this temporal feature with respect to the entry. However, considering the high correlation coefficient (r = 0.856) of HEAD_EXIT_, the results of both features revealed an accuracy sufficient for analysis purposes. Indeed, the error value of HEAD_EXIT_ expressed in percentage of the stroke cycle duration was, in any case, lower than previous studies investigating temporal stroke features. Specific comparison with existing literature could not be performed, as the present study was the first to validate an algorithm for timing breathing detection using IMUs. However, it has been demonstrated how investigating the breathing action in front-crawl swimming is fundamental not only to the energetic efficiency, but also to the effect on upper limb kinematics and overall technique [28]. For this reason, a reliable algorithm for the detection of breathing timing allows monitoring of the effects of fatigability or different techniques on the breathing timing, in addition to the intratrial variability of these effects.

Head and upper/lower limb actions of the swimmer during front-crawl swimming were accurately and repeatably identified in terms of absolute temporal value (Figure 3). Furthermore, the stroking, kicking, and breathing features were integrated with the stroke cycle duration (Table 1 and Figure 4), allowing a reliable combined timing analysis of the action of all principal factors in front-crawl using the minimum number of inertial sensors. Swimming is a cyclic activity, and the synchronization between the head and upper/lower arms can only be optimal if it can be maintained along consecutive stroke cycles [23]. In order to produce the propelling effects, the stroking, kicking, and breathing must occur at the same relative time in each stroke. The integrated timing analysis proposed in this study could enable swimming coaches to continuously check the whole timing technique of the swimmers during the training session.

The accuracy and repeatability findings must be considered reliable for the motor task and level of swimmer analyzed. Further investigations will explore the generalization of the results for different coordination models of technique, performance level of swimmers, and different swimming techniques. It must also be highlighted another limitation in the stroking was identified by the entry of the wrist into the water, as the IMU was attached to the wrist. Thus, with the aim of limiting the swimmer burden, the catch event more strictly related to propulsion was not possible to be detected. Coaches could take advantage of the proposed tool to continuously check the whole timing technique and monitor the effects of fatigue or different techniques on the coordination timing.

In conclusion, a protocol for integrated analysis of stroking, kicking, and breathing using inertial sensors in front-crawl swimming was developed and validated in comparison with a video-analysis technique. All accuracy parameters investigated (RMSE, bias, LoA, and correlation) highlighted high agreement with the gold standard. Furthermore, the protocol proposed was user-friendly and as unobtrusive as possible for the swimmer, allowing a stroke-by-stroke analysis during the training session.

## Figures and Tables

**Table 1:** Mean ± SD timing of the analyzed temporal events in percentage of the stroke duration when comparing measurements with IMUs and video-based technique (TLC). The stroke duration was normalized to left WRIST_ENTRY_ events.

Temporal Events	IMUs	TLC
First right LEG_DOWNBEAT_ (%)	2.3 ± 7.6	3.1 ± 6.9
First left LEG_DOWNBEAT_ (%)	15.5 ± 9.4	16.0 ± 9.3
Right HEAD_EXIT_ (%)	15.9 ± 5.7	18.7 ± 2.8
Right HEAD_ENTRY_ (%)	48.0 ± 2.0	48.4 ± 2.0
Right WRIST_ENTRY_ (%)	49.3 ± 2.2	49.0 ± 2.1
Second left LEG_DOWNBEAT_ (%)	53.7 ± 8.3	53.0 ± 7.6
Left HEAD_EXIT_ (%)	58.2 ± 3.5	65.2 ± 3.5
Second right LEG_DOWNBEAT_ (%)	64.1 ± 8.2	64.8 ± 8.7
Left HEAD_ENTRY_ (%)	96.6 ± 1.0	96.8 ± 0.6

## Data Availability

The data presented in this study are available upon request from the corresponding authors. The data are not publicly available due to privacy restrictions.
